# Supplementary figures and images for: Mapping resistance-associated anthelmintic interactions in the model nematode Caenorhabditis elegans
Source: PLoS Negl Trop Dis. 2023 Oct 26;17(10):e0011705. doi: 10.1371/journal.pntd.0011705 (PMC10629664; doi:10.1371/journal.pntd.0011705)

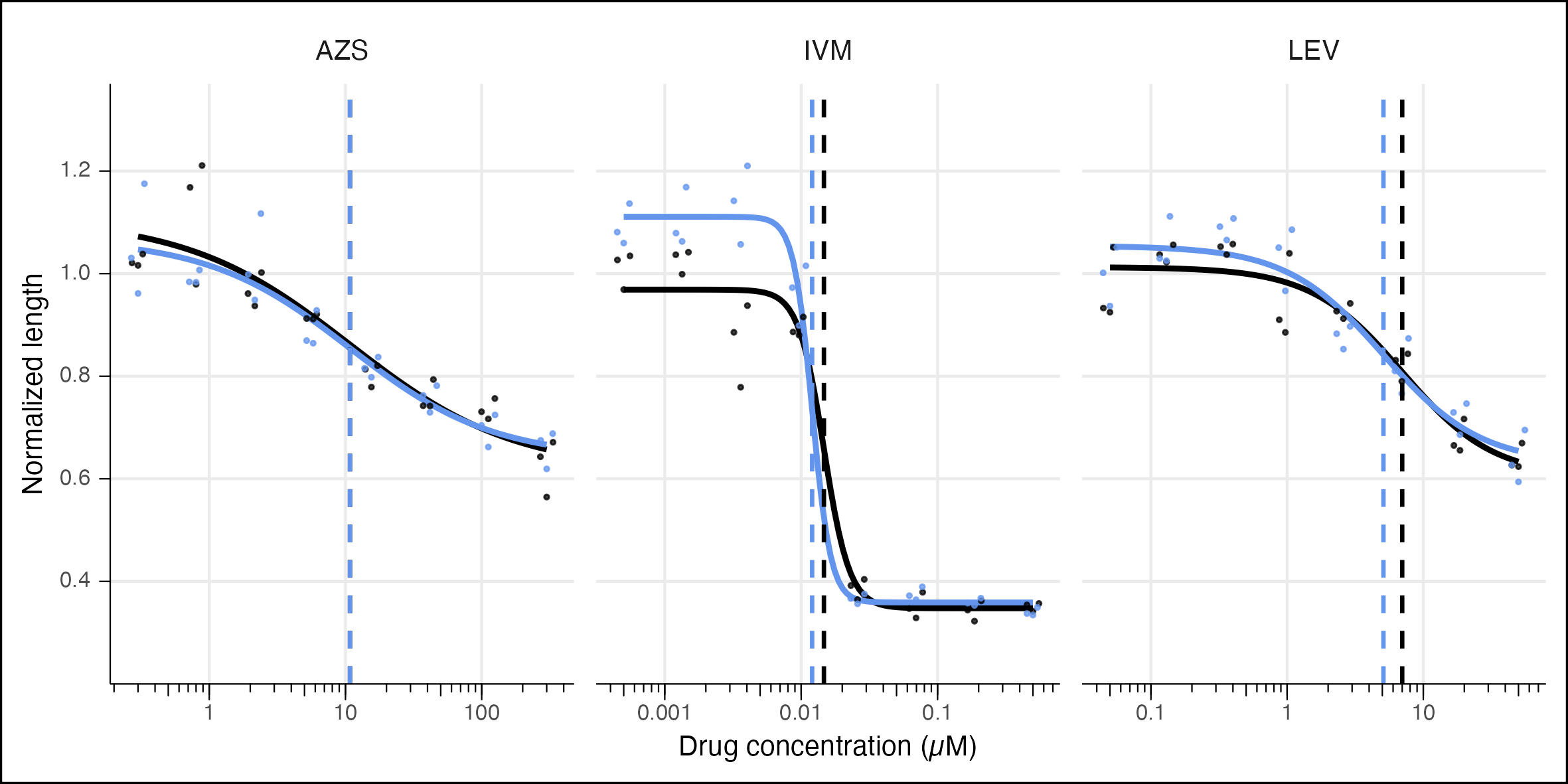

Supplement: S1 Fig — Dose response data is shown for eggs hatched on unseeded NGM plates (black) and in glass tubes (blue). (TIFF) [file pntd.0011705.s001.tiff]

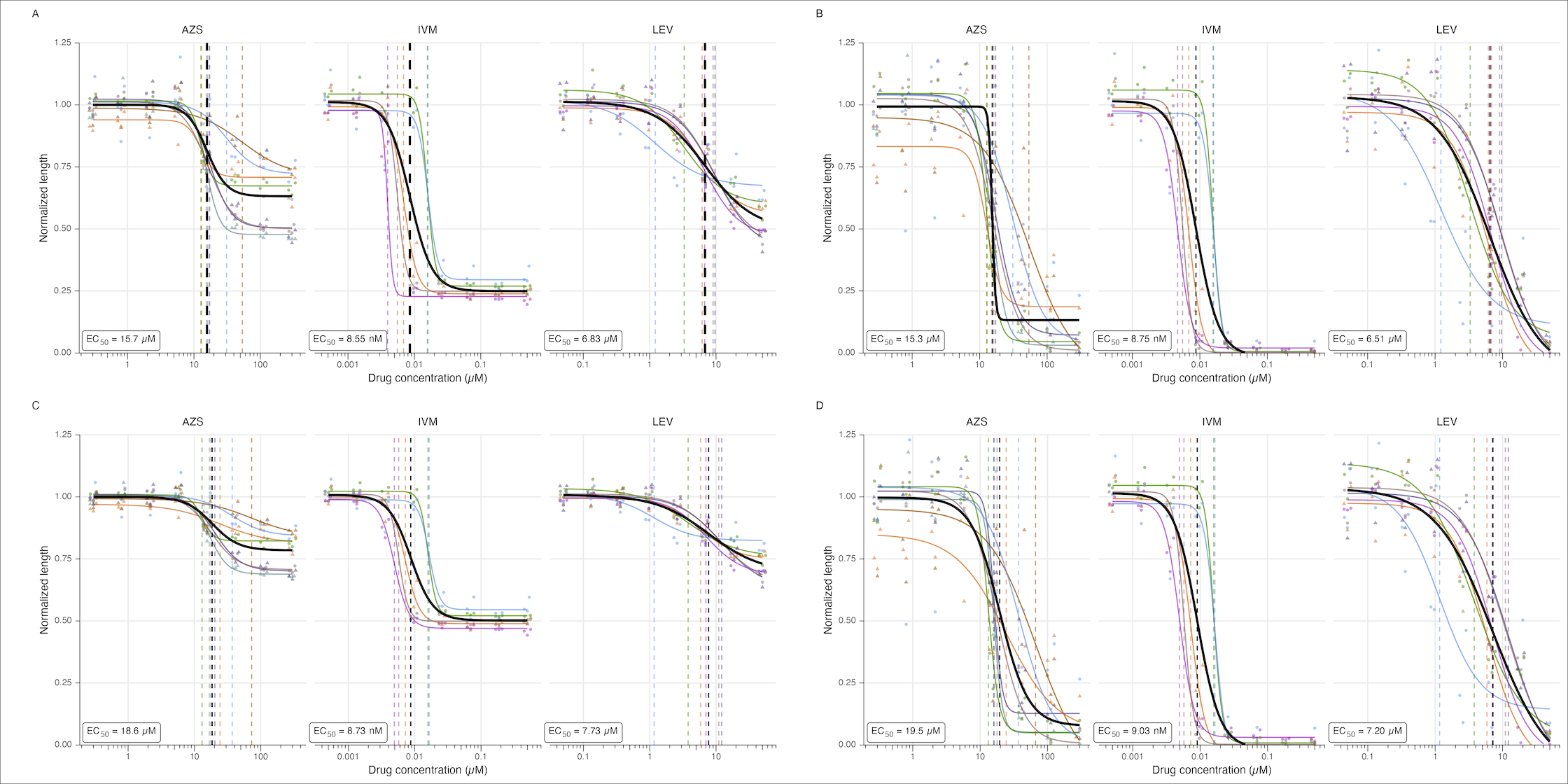

Supplement: S2 Fig — Analysis was performed considering four normalization schemes, and patterns and curves of best fit vary minimally between methods. A) Phenotypic data was normalized by dividing individual values by the average of the control (1% DMSO). B) Max-min normalization using the highest concentration of drug as the minimum and the DMSO control as the maximum. C) The normalization procedure in (A) preceded by a square root transformation. D) The normalization procedure in (B) preceded by a square root transformation. (TIFF) [file pntd.0011705.s002.tiff]

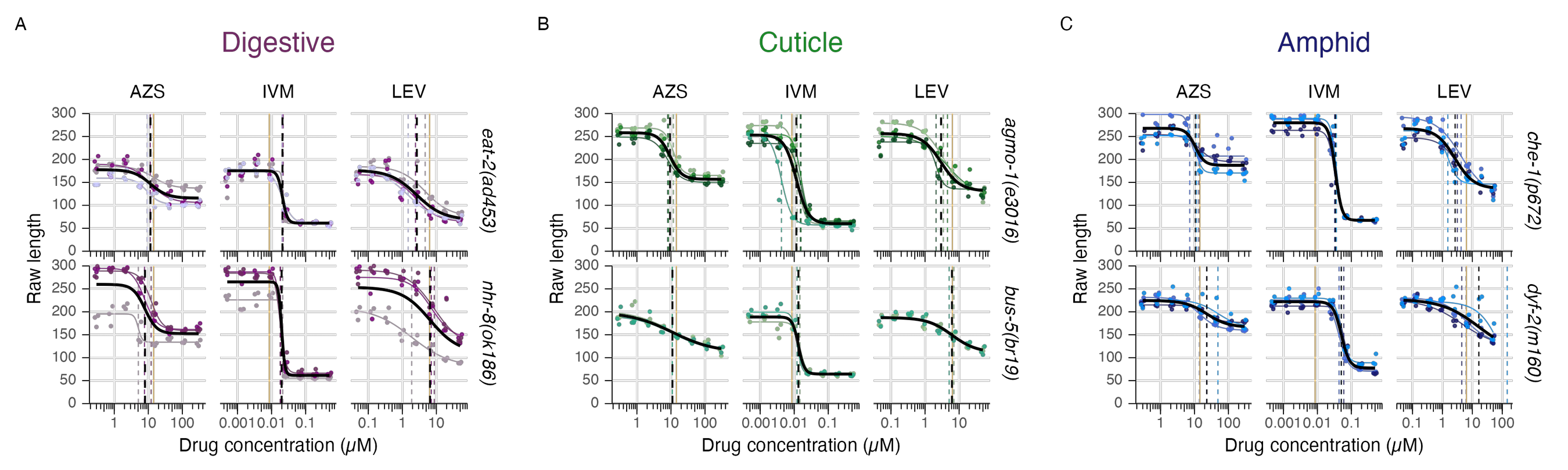

Supplement: S3 Fig — Curves were produced by inputting the raw output from wrmXpress. Normalization by dividing values by the average of the control group from the corresponding biological replicate does not affect the inferences of hypersensitivity or increased resistance. (TIFF) [file pntd.0011705.s003.tiff]

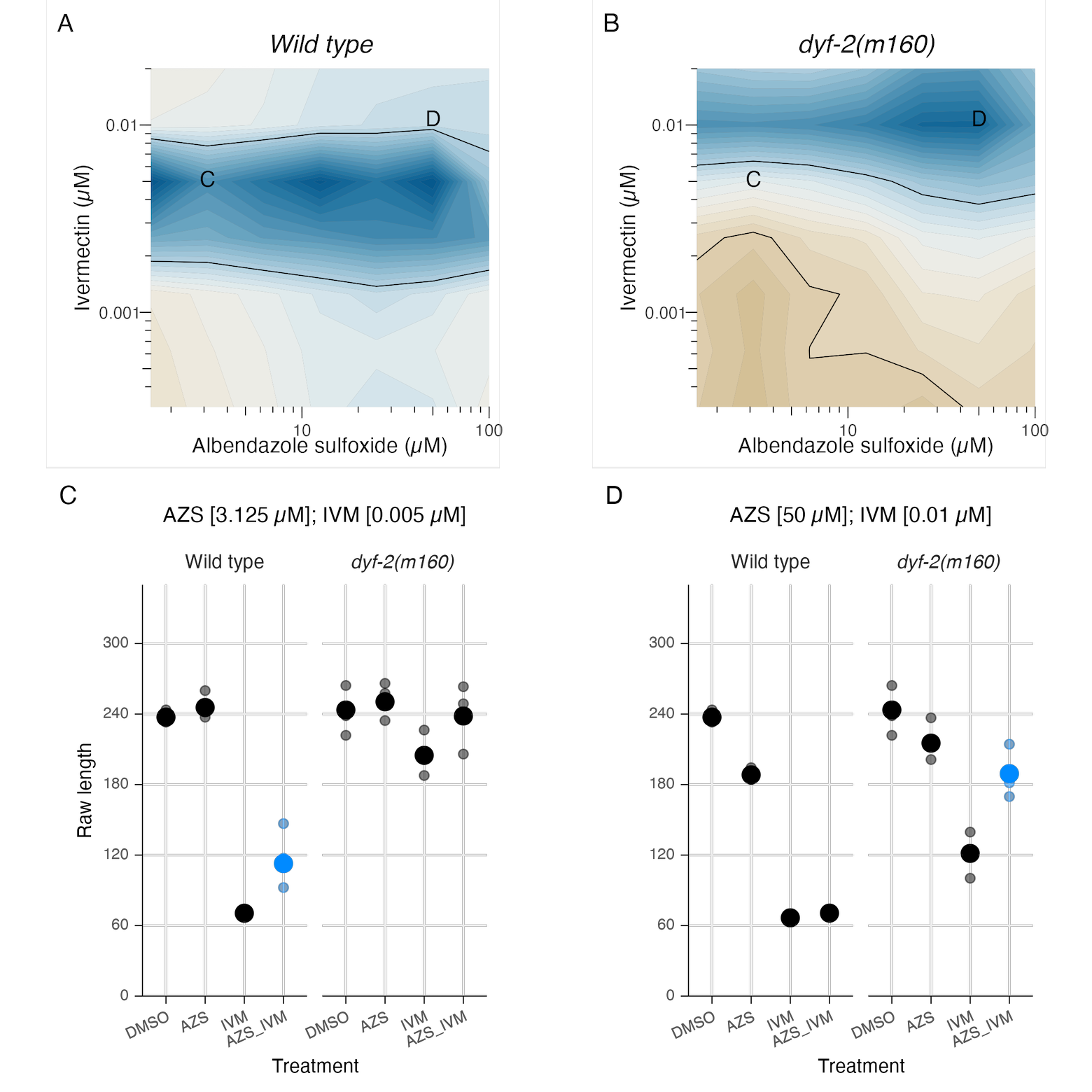

Supplement: S4 Fig — A-B) Synergy and antagonism contour plots of wild type and dyf-2(m160) copied from Fig 4 with letters labeling the combinations of doses plotted in C and D. C-D) Raw lengths plotted at two different concentration combinations for the control group (DMSO), AZS alone, IVM alone, and AZS and IVM combined. The wild type strain shows antagonism at 0.005 μM IVM and 3.125 μM AZS (represented in panel C with a blue dot), while the dyf-2 strain shows no interaction between AZS and IVM at these concentrations. The dyf-2 strain shows antagonism at 0.01 μM IVM and 50 μM AZS (represented in panel D with a blue dot), while the wild type strain shows no interaction between AZS and IVM at these concentrations. (TIFF) [file pntd.0011705.s004.tiff]
